# A Data-Driven Intelligent System for Assistive Design of Interior Environments

**DOI:** 10.1155/2022/8409495

**Published:** 2022-08-25

**Authors:** Guoxing Chen

**Affiliations:** College of Fine Arts, Guangdong Polytechnic Normal University, Guangzhou 510665, Guangdong, China

## Abstract

This paper analyses the design of a healthy interior environment using big data intelligence. The application of big data intelligence in the design of healthy interior environments is necessary because the traditional interior design approaches consume a lot of energy and other problems. Benefited by its strong ability of computation and analytics, artificial intelligence can well improve a series of problems in the field of interior design. The proposal summarizes the sources, classifications, and expressions of behavioral data in interior spaces, carries out analysis and research on behavioral data from two aspects: display space and supermarket space, summarizes the interior methods based on behavioral data, and analyses the visualization application of behavioral data in different interior scenes, to explore the application value of behavioral data in interior design. In contrast to it is the unconscious behavioral response, the biggest characteristic of which is that it is regulated by the behavioral subject's physiological factors or habits of the behavior issuer. In this paper, we convert the layout recommendation problem of a space into a functional classification problem of segmented segments and household segments on a plane. The scene layout features are extracted by binary coding, the abstraction of the cross features between the vector segments is achieved by using a word embedding algorithm, the feature matrix is reduced in dimensionality, and finally, the segmentation network model and the layout network model are constructed, respectively, by using a bidirectional LSTM. The experiments show that the accuracy of the layout recommendation model in this paper is 98%, which can meet the demand for real-time online layouts.

## 1. Introduction

In today's rapidly developing society and technology, people's life is becoming increasingly fast paced, and those who have been working all day, need a convenient and comfortable interior design to meet their needs under extremely exhausting conditions [1]. The integration of AI intelligence into the interior design will improve some of the shortcomings and deficiencies of current interior design, maximizing the needs of occupants, bringing more convenience and safety to people's daily lives, and minimizing energy consumption. For a long time, the original interior design concept of modern housing was created by the rationalization of room layouts and the convenience of interior lighting. More attention has been paid to the extent to which the objective hardware matches and only a few designers have taken the initiative to focus on the type of environment in which the inhabitants live, but this is the only way in which they can truly experience the comfort and pleasure of everyday life. Home designs that overlap with more interior decoration can accidentally cause damage while requiring more time and more difficult cleaning, which in turn makes residents regret having chosen this type of interior design and often worry about it [2]. The model of the next stage is constructed based on the model of the previous stage, and the construction of the model of the previous stage should be considered for the construction of the next stage. These types of interior furnishings are beautiful from the resident's point of view but certainly, add to the many potential problems from the feeling of the experience. In this way, the interior design has little connection to the inhabitants, and then the existence of the interior design is not important to the inhabitants, which is extremely detrimental to the development of the interior design.

AI intelligence falls under the umbrella of computer science, which aims to create intelligent machines using human intelligence. The scientific revolution is one of the main reasons for the emergence of AI intelligence and is one of the keys to its technological development. Scientific research suggests that AI intelligence has emerged to effectively stimulate people's minds, understand themselves, and change the world. In the field of AI intelligence, attitudes toward AI intelligence research are different, but the overall performance is in favor of creating strong AI intelligence with the same level of thinking and understanding as humans and in favor of creating weak AI intelligence with incomplete information. AI intelligence is based on the combined behavior of systems research, psychology, control technology, computer technology, and sound technology [3, 4]. In addition, various mathematical models and theories are needed to increase the speed of AI intelligence. Today, the development of AI intelligence technology is mainly seen in facial recognition, autonomous driving, and banking data systems. The three main technologies that provide AI intelligence are common programming methods, machine learning methods, and learning methods[5, 6]. In modern society, entertainment and recreation are an important part of people's activities. The advancement of urbanization and the improvement of people's quality of life have led to a tendency for cinema spaces to focus on people's own experiences, promoting design that keeps pace with the times and focuses on the human experience, promoting interaction between people in their activities, mainly the formation of interactions between people, objects, and the environment.

Currently, some people are adding one or more home cleaning robots to their homes to enhance the comfort of rest and living and to regularly clean dead areas that are difficult to clean manually, for overall cleaning. However, for a significant segment of the robots on the market, this cleaning is only superficial, i.e., the quality of the cleaning depends on the exact position of the furniture and appliances. The functional spaces that are related to each other and close to each other are divided into the same model file, which not only meets the design requirements but also realizes the effective use of resources. The cleaning path is created and works according to the installed route, with the disadvantage that it cannot be disturbed by the outside world while working. If a person is in the same room as the cleaning robot, he or she should carefully avoid it so as not to interfere with the robot's normal work, but this can affect people's daily experience. In addition to this, very few indoor appliances are in an intelligent state, which shows that the current application of artificial intelligence in the field of interior design still needs to be improved and popularized. Therefore, the introduction of a new multiorder modulation and demodulation technology in indoor multicolor multiplexed VLC systems, and the integration with commonly used fiber optic communication systems, can effectively improve the system transmission rate and band utilization, and reduce the cost of transceiver consumption. Based on the above considerations, it is of great theoretical and practical significance to research modulation and demodulation techniques for multicolor multiplexed visible light signals for indoor big data access.

## 2. Related Works

Behavior generates behavioral data, and behavior data are a quantitative representation of behavior. Behavior is diverse, so there are differences in the corresponding behavior data. Based on the above research results, this thesis classifies behavior data into two main categories: location data and experience data [7, 8]. Location data refers to objective data in life, mostly monitored or uploaded by mobile phones, radio frequency identification devices, GPS, and other devices, including the location of the user, the trajectory line data of individual movement, the surface data of group movement, and the related thermal data [9]. Experience data include user evaluation and feedback data, which is highly subjective and is mainly collected from web pages and mobile clients. The research in this paper focuses on the location data of user behavior data. Indoor location data contain rich and complex behavior information, which provides new research ideas for interior design innovation, so that design relies on scientific and rigorous data analysis, rather than relying on previous empirical talk. Serrano et al. address the problems of low electrical energy utilization and waste in library lighting systems by analyzing the data collected from seated readers and designing a zoning lighting system that meets the behavior habits of readers while saving resources and reducing waste [10]. The research results were successfully applied to the secondary planning and renovation of the commercial street by using behavior models and computer simulation methods and based on the virtual visitation data of the online Expo, they obtained the behavior data of the visitors and carried out the temporal and spatial simulation of the multi-individual visitation behavior through the model, achieving the prediction and guidance of the number of visitors, the flow of people and the demand for facilities at the Expo [11]. The average accuracy of a single unit segment of the layout model without the word embedding layer is calculated to be 89.75%, and the average accuracy of the entire sequence of units is 73.48%; the average accuracy of a single unit segment of the layout model with the word embedding layer added is 98.60%. The model is used to predict and guide the number of visitors, and the demand for facilities, and to visualize the spatial and temporal changes in the pavilion.

Rosário et al. proposed an improved Pearson collaborative filtering algorithm, which uses a prediction model calculated from user profiles, item characteristics, and user behavior data instead of the similarity calculation of traditional algorithms, optimizing the traditional recommendation algorithm [12]. Hong et al. proposed an improved CF recommendation algorithm using singular value decomposition to achieve dimensionality reduction, which can alleviate the sparsity problem of the scoring matrix and achieve better recommendation results [13]. Barbar et al. proposed a deep learning-based multi-criteria collaborative filtering model, which takes the characteristics of users and items as inputs to a standard rating deep neural network, and the prediction results of the first part form the inputs to the second part of the overall rating deep neural network [14]. The study of this model provides a basis for using deep learning and multi-criteria in recommender systems [15].

As mentioned above, content-based recommendation algorithms need to know information about 3D home models, 3D layout scenes, etc. Although there is no project could start problem, the extraction process of its features is difficult and computationally complex; a single collaborative filtering algorithm will have problems such as matrix sparsity and cold start when solving the intelligent design problem studied in this paper; association rules and popularity recommendation algorithms are effective in the cold start problem. However, there are still some shortcomings in achieving fast and accurate recommendation effects. Because of this, to better solve the recommendation problem faced by this paper, inspired by many recommendation algorithms, this paper is based on a hybrid recommendation algorithm, taking the strengths of each, dividing the intelligent design process into two parts: home matching and home layout, designing different recommendation models, respectively, and combining the two recommendation models utilizing transformation, triggering the corresponding recommendation model according to the user's needs.

## 3. Artificial Intelligence in Interior Environment Design Application under Big Data Environment

### 3.1. AI Analysis of Big Data for Indoor Environments

The rapid development of the Internet has catalyzed the advent of the era of information data explosion, and the emergence of massive information data has exacerbated the problem of information overload. To effectively alleviate this pain point of information data overload, recommendation algorithms have shown their great charm by analyzing the behavior characteristics of users and the attributes of items to obtain effective recommendation data, and then provide more meaningful recommendation services for users [16]. Grid floor plans are not optimal for retailers who want to create an upscale branded environment, which can cause customers to browse easily and not be impressed with the merchandise. A model-based recommendation algorithm is a typical machine learning problem in which historical data of a research object is used as a training sample, a network model is defined to describe the potential recommendation relationship between the research object, and model parameters are obtained through an optimization process, and an appropriate prediction model is trained to achieve recommendations.

The matching recommendation model in this paper is designed for the matching of 3D home models. Considering the professional nature of interior matching solutions, the aesthetics of ordinary users, and the limited matching data make it difficult to construct a recommendation model with good recommendation results, so user-based recommendation solutions are discarded. In this paper, professional matching schemes are selected as the data set and the matching correlation between items is used to build a basic matching recommendation model:

(1) $$\begin{array}{c} Q=\int_{280nm}^{720nm}683\frac{lu}{W}S\left(\kappa^{2}\right)v\left(\lambda \right)\text{d}\lambda . \end{array}$$

To improve the efficiency of the intelligent design algorithm, this chapter investigates the matching recommendation algorithm for 3D home models in the context of an interior space design software platform. The basic collocation recommendation model is constructed using the item-based collaborative filtering recommendation algorithm [17]. To improve the computational efficiency, the two-dimensional rendered image of the home model is taken as the processing object, the features of the image are extracted by the convolutional neural network, the feature vector library of the image is constructed, and the ideas of the content-based recommendation algorithm are mixed to populate the highly sparse collocation recommendation matrix to realize a new hybrid collocation recommendation model:

(2) $$\begin{array}{c} I\left(\phi \right)=\frac{I_{0}\left(\phi \right)}{d}. \end{array}$$

In this paper, 846-bedroom home matching solutions paired with professional designers were selected for experiments on the interior space design software platform, which contained 5 home item categories with a total of 1240 home items, 190 TV cabinets, 418 beds, 257 bedside tables, and 201 wardrobes. In total, 600 randomly selected matching data were used as the training set to build the matching recommendation model, and 300 randomly selected data were used as the test set from the 712-test data constructed from the remaining 246 data expansions to test the accuracy of the recommendation model. A set of matching solutions can be represented as a five-tuple (TVark_14, bed_149, bedtable_12, dresser_1, wardrobe_1), and each household item can be represented as where “type” is the type name of the item and “num” is the type number of the item. Therefore, each matching scheme can be represented by a five-tuple, where 0 means that the corresponding household item in the scheme does not exist (Table 1).

The correlation of design information in this row refers to the interrelationship between various information and components, e.g., nongeometric information needs to be represented by geometric information as a carrier, and the description of the performance of geometric forms needs to be represented by nongeometric information. In the process of using information models to describe the interior design, the information model is used in the design expression, design and construction, and operation and maintenance phases [18]. Therefore, the user-based recommendation scheme is abandoned. This paper selects the professional collocation scheme as the data set and uses the collocation correlation between items to construct the basic collocation recommendation model. The description of interior components in the information model is based on real building components and has the same building properties and interrelationships between components as reality.

Doors and windows are dependent on walls and cannot be created without the existence of walls, i.e., walls are the subject of the existence of doors and windows; there is a spatial distance between the floor and the ceiling, etc. The logical interconnection between this information forms the spatial use ecosystem of the interior information model.

(3) $$\begin{array}{c} S\left(\phi \right)=\left\{\begin{array}{cc} \frac{m+1}{2\pi }\sin^{m}\left(\phi \right), & \phi \in \left[-\frac{\pi }{2},\frac{\pi }{2}\right],\\ 0, & \text{others.} \end{array}\right. \end{array}$$

**Figure 1:**
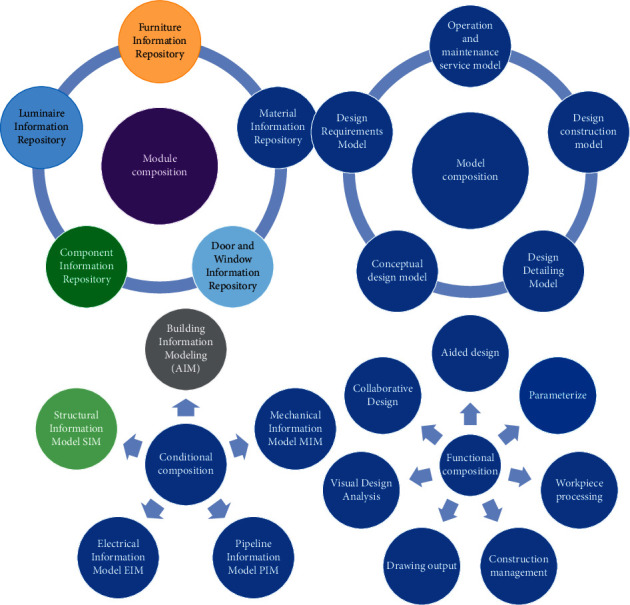
Diagram of the interior information model.

Combining the definition of BIM and the characteristics of interior design itself, I believe that the interior information model is based on the information models of architecture, structure, and electromechanical pipelines and consists of a library of various resources related to interior design, which can assist in design, design collaboration, visual design, parametric design, and other functions, throughout the design stages such as scheme design, deepening design and construction drawing design, and can be a 3D visual information model for drawing output (effect drawings, scheme drawings, effect drawings, etc.), construction management, production and processing of decorative components, etc. The interior information model enables the management of the life cycle of interior spaces [19]. The model is based on design information, and by integrating the information associated with it, it forms a database that can be used to manage and maintain the entire life cycle of interior space (Figure 1).

In simple terms, an interior information model is an overview of the interior design process, an abstract understanding and representation of the form, composition, and materials of the interior space, and the manufacture, construction, and management of the building components. The features of the image are extracted by the convolutional neural network, the feature vector library of the image is constructed, the idea of a content-based recommendation algorithm is mixed, and the highly sparse matching recommendation matrix is filled to realize a new mixed matching recommendation model. An interior information model in its fullest sense should contain two related aspects: a spatial geometry model and design data information. The spatial geometry model reflects the relationship, form, and composition of the interior space and is the carrier of the design data information; the design data model reflects the nongeometric information related to the spatial geometry model, including technical requirements and design parameters.

(4) $$\begin{array}{c} g\left(\theta \right)=\left\{\begin{array}{cc} \frac{m^{2}}{\sin^{m}\left(\theta \right)}\sin^{2}\left(\theta \right), & \theta \in \left[-\frac{\pi }{2},\frac{\pi }{2}\right],\\ 1, & \text{others.} \end{array}\right. \end{array}$$

The result of using BIM in interior design should be that all designers apply it to the whole design process. However, at this early stage of application, the conditions for full application are not yet available, and the application of partial stages and partial processes will be a norm for the application of interior information models.

According to the division of specific project design requirements, design process and design stages, different stages of model construction can be selected in the process of interior information model construction. From a macro perspective, the life cycle of an interior space is divided into five stages: requirements analysis, conceptual design, design deepening, design implementation, and maintenance support [20]. The function of the information model is in the stages of design expression, design and construction, operation, and maintenance, etc. According to the divided stages, design demand models, conceptual design models, design deepening models, design construction models, and operation and maintenance service models can be built, respectively, while the conversion, integration, and operation between the models of each stage need to be organically realized.

**Figure 2:**
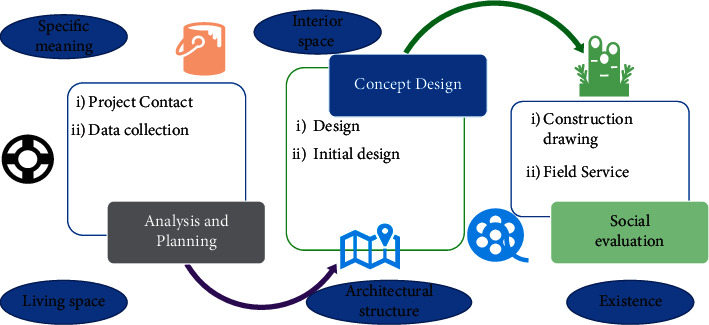
The data realization process.

On the one hand, in terms of specific meaning, the understanding of interior space is very different from the understanding of space in other fields, it is formed according to the modern living space to divide, has the function of enclosing the architectural structure, and is formed by a real substance to form an objective existence. On the other hand, the design of four-dimensional space incorporates the way people live and behave, making it possible to use time as a primary measure in this new spatial system, giving space, which does not have any qualities in the first place, an external substance and time, so that space has its value of existence in this dimension. All these manifestations culminate in a spatial essence with its characteristics, as shown in Figure 2.

Based on the relevant project information, the first step is to carry out a design analysis. Design analysis is the partial reverse operation of design thinking, the purpose of which is to extract as much information as possible about design thinking from local or limited material, and to synthesize, summarize and refine it through the analyst's thinking so that it becomes an important part of his or her design ideas, techniques, and approaches.

Interior space is variable, and changes to the physical interior, such as changing the location of a home or removing or adding new walls, can actively or passively change the structure and connectivity of the interior space [21]. The description of the interior components by the information model is based on the real building components, and has the construction attributes consistent with reality and the interrelation between components. It is because of this variability that people can personalize the design of interior spaces to meet their individual needs. Indoor spaces are generally private, which is a significant difference from publicly shared outdoor spaces. Private spaces are designed to better protect the privacy of the individual so that the individual can be free from the outside world, and free from the influence of the outside world. Certain public spaces also have a certain degree of privacy, such as warehouses or self-service withdrawal areas in banks, which are open to specific people at specific times.

### 3.2. Artificial Intelligence in the Design of Interior Environment Applications

Behavior is generated by a variety of physical and psychological needs. There are a variety of behavioral responses to external information, but these can be broadly divided into conscious and unconscious responses. Conscious reactions are widespread in people's daily lives and come from their control, often with a strong sense of purpose [22]. In contrast, there are unconscious reactions, which are characterized by the physiological factors or habits of the subject of the act, which wield the behavior of the emitter.

The geometric information is highly visible and includes both the visual presentation of geometric forms and the two-dimensional planar relationships of drawings. This collaborative working process brings together information from within and outside the various disciplines to synthesize and communicate information in the same information model, i.e., through a single design medium.

**Figure 3:**
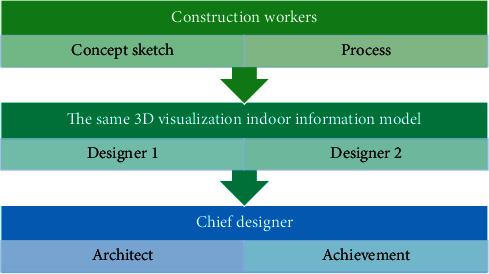
Schematic diagram of interior design collaboration centered on the information model.

As can be seen in Figure 3, the collaboration between team members throughout the process, from design conception to design expression to design implementation, is based on the same information model, through which information is obtained and transferred between team members. In this process, the geometric and nongeometric information of the design is transferred through the visual information model, with a high degree of visibility of the geometric form. The model is based on design information, and at the same time, by integrating the information associated with it, a set of data information bases that can be used to manage and maintain the entire life cycle of indoor space is formed. At the same time, information transfer and communication between design and construction are based on the visual information model, which has both three-dimensional geometry and two-dimensional drawing lines.

Unlike CAD, which is a geometric description of the design object, the information model is a description of the characteristics of the design object, and the geometric modeling and information description of the space for each stage of the different characteristics, resulting in different descriptive models, which are used throughout the life cycle of the space, connecting all aspects of the space from planning to use and even redesign, creating a virtual space that is consistent with the real space.

The phasing principle is based on the model deepening method to construct the model in stages: design requirements stage, conceptual design stage, design deepening stage, construction design stage, and operation and maintenance stage, each of which has a different level of maturity for the design model. In the process of constructing an interior information model, different stages of information models are constructed according to the different levels of information generated at different stages [23]. To provide users with more meaningful recommendation services. Throughout the design process, the design model of the interior information model will include the design requirements model, the conceptual design model, the design deepening model, and so on.

The design requirements model is mainly used to model the user's needs, by analyzing and collating the user's design requirements for the interior space, the user's behavior habits, and other factors, the user's needs information is transformed into design information. The conceptual design model is a conceptual arrangement and planning of the design through the analysis and synthesis of the user's demand information, mainly based on the geometric information of the space, including the spatial shape, spatial relationship, environmental analysis, and other content. The design deepening model is based on the conceptual design model, further quantifying the information of various design parameters, verifying the design scheme, forming a clearer design scheme, and preparing for the construction model construction.

**Figure 4:**
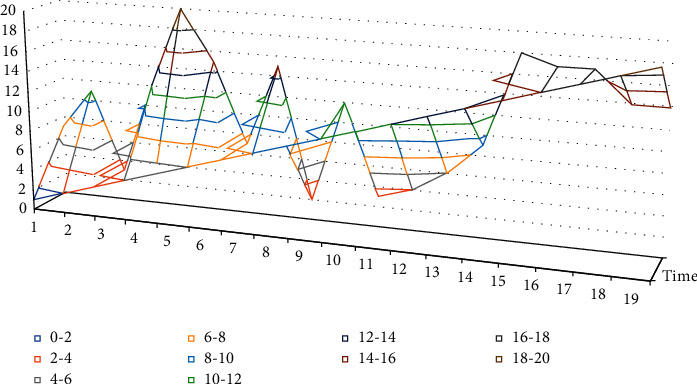
Time-domain waveform of the signal.

The principle of model layering refers to one of the ideas on how to solve the problem of increasing the amount of information in the model, model layering is achieved through the division of the model. It is necessary to divide the model of the whole project into several relatively independent files based on the design task requirements, functional zoning, etc. and to integrate and link the various parts of the files after the model is constructed, as shown in Figure 4.

Since there are relatively few links between floors and the upper and lower floor spaces do not affect each other, dividing the model files according to floors is the commonly used layering approach. According to the divided stages, the design requirement model, conceptual design model, design deepening model, design and construction model, and operation and maintenance service model can be established, respectively. However, if there is an overhead space, which is often the outstanding part of the design, it is no longer appropriate to divide the space according to the floor area, so it is necessary to divide it according to the functional area of the space and divide the functional spaces that are close to each other into the same model file, to meet the design requirements and achieve the effective use of resources.

In interior design, the choice of decorative materials is a fundamental issue, and without the appropriate tailoring support, the best spatial design concepts cannot be fully expressed. Before selecting decorative materials, it is necessary to grasp the characteristics of the different materials and the context in which they are used [24]. Virtually every material in the world can be used as a decorative material, but the range of materials chosen is directly related to the type of material known. The best way to have better creativity is to broaden your knowledge of decorative materials and to have a thorough and systematic understanding of new materials. Again, good materials can sometimes lead to better creativity and ideas. About the understanding of the concept of good materials, many people think that expensive materials are good materials, but they are not. The goodness or badness of materials is usually determined by the designer's design requirements, for different situations, and for individual interior design, material innovation is also important.

## 4. Analysis of Results

### 4.1. Performance Results of Artificial Intelligence Algorithms for Interior Environment Big Data

**Figure 5:**
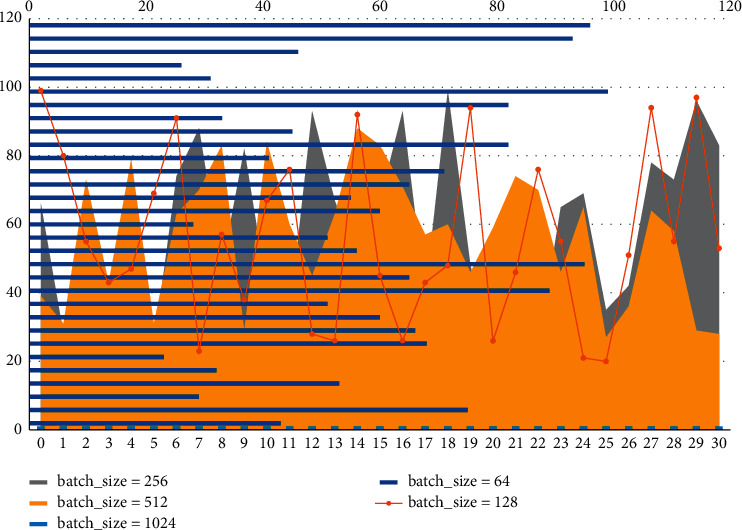
Layout network model comparison experiment.

The structure of the layout network model is like that of the segmentation network model, and the parameter adjustment process of the layout network model is used here as an example to analyze the parameter determination method during the experiment in detail. It will actively or passively change the structure of the interior space and the connectivity of the interior. The training process uses the batch method to split the whole dataset into different small subsets, using these small subsets to complete a forward and backward iterative process, making full use of computer resources. Different values of batch size were used for comparison experiments, and the curves of accuracy and loss of the training set with the number of iterations were obtained as shown in Figure 5 below. The following curves were analyzed, and the best accuracy rate could reach 100%, while the size of batch size would affect the training speed, and considering the smoothness of the curve and the training speed, the batch-size size of 128 was chosen for this paper.

**Figure 6:**
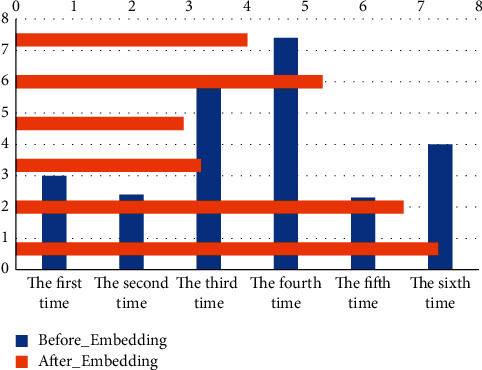
Test set accuracy.

This experiment uses 3000 household layout data to test the accuracy of the layout network model. The accuracy of individual household segments refers to the prediction accuracy of a single segment in the sequence describing the household information, and for the whole sequence of household segments, if one of the segments is incorrectly predicted, the prediction result of the sequence of household segments is inaccurate and the value is 0. The scene layout features are extracted in the form of binary coding, and the abstract extraction of cross features between vector segments is realized by a word embedding algorithm. The average accuracy of a single segment of the layout model without the word embedding layer was 89.75% and the average accuracy of the whole sequence of segments was 73.48%; the average accuracy of a single segment of the layout model with the word embedding layer was 98.60% and the average accuracy of the whole sequence of segments was 92.16%. Embedding network model and the Before_Embedding network model are shown in Figure 6, the average test speed of the After_Embedding network model is 17.88 s, and the average test speed of the Before_Embedding network model is 309.43 s.

The layout network model with the word embedding layer learns the features between scene information and layout better, and its performance in the test set is significantly better than that of the Before_Embedding model; comparing the average prediction accuracy of individual household segments and the whole sequence of household segments under the same layout network model, the After_Embedding model has a higher accuracy of the whole sequence of household segments than that of individual household segments. The sequential accuracy of the After_Embedding model decreased by 6.53% compared to that of the individual household segments, while the sequential accuracy of the Before_Embedding model decreased by 18.13% compared to that of the individual household segments.

But only in this way can the occupants truly experience the comfort and joy of daily life. This is because the features characterized by One-hot coding are independent of each other, which ignores the similarity and correlation between coded segments, while the word embedding layer can more reasonably express the relationship between the size and distance of coded segments, which is the abstraction of the extraction between features and the expression of the potential relationship between coded values. In addition, the word embedding layer thickens the One-hot matrix and reduces the dimensionality of the feature matrix, which greatly improves the prediction speed of the model.

Considering that the layout time of the distance field algorithm and the placement field algorithm is densely related to the number of models to be laid out in the scene, here 1000 randomly selected data to be laid out are used to evaluate the algorithm performance in terms of their total layout time. It should be added that the running times of the distance field layout algorithm and the placement field layout algorithm in Figure 6 are estimated based on the experimental results of the algorithm proponents, and the time of the layout algorithm in this paper is the sum of the segmentation and layout times. The comparison leads to the conclusion that the layout algorithm in this paper has better performance in terms of real-time processing.

### 4.2. Analysis of the Results of the Application of Artificial Intelligence in the Design of Indoor Environments

The artificial intelligence environment can monitor every electrical appliance in the home in real-time, so that not only can the waste of resources be reduced, but also avoid accidents, for example, the power can be cut off in time or when there is a fire can be implemented automatically in the whole room in time to put out the fire, and other dangerous situations can be strongly avoided, so that it brings a higher level of security to people's living. It also provides a certain level of security for the elderly and children.

The inhabitants of an artificially intelligent environment can turn many electronic devices on and off at any time, depending on when they leave home in the morning and when they return home in the evening. When leaving the house in the morning, appliances such as lamps, microwave ovens, and air conditioners can be switched off automatically, which reduces unnecessary electricity bills; when returning in the evening, essential items such as lighting and air conditioning are automatically set to recognize the occupant and turn on automatically, thus reducing the tedious process of manually setting switches and providing a humane living environment for a weary worker.

In the specific construction process of interior space environment design, and new construction solutions, designers are often excited by their bold ideas, but we cannot try and verify whether they are correct. The traditional implementation plan inevitably has errors in the construction data, then, the design plan draft and the construction of the handover link must have a professional engineer to do guidance, according to the construction site to do size adjustments and modifications. In this way, the interior design has little connection with the occupants, so for the occupants, the existence of the interior design is not important, which is extremely unfavorable to the development of the interior design. The corresponding construction plan is verified in time. The use of digital technology allows for more precise positioning of the design, while the specific construction plan can be demonstrated through three-dimensional technology, simulating the impact of environmental factors on the construction, to develop a feasible construction plan and verify the feasibility of the finalized construction plan. This improves the efficiency of construction and shortens the construction cycle.

**Figure 7:**
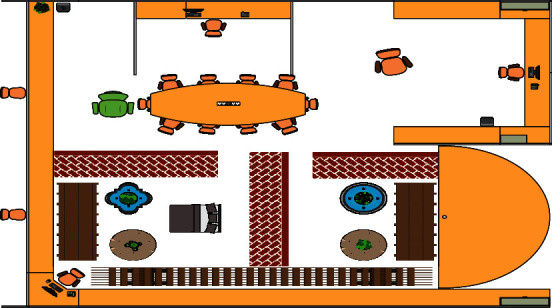
Layout diagram.

As a kind of art closely related to human lifestyle, interior environmental art design, in the era of rapid development of digital technology, new means of design are emerging, and traditional design procedures and methods can no longer adapt to the current needs. All make the cinema space tend to pay attention to people's own experience, promote the design to keep up with the pace of the times, take people's experience as the core, and promote people to interact in activities, mainly the formation of the interaction among people, objects, and the environment. It will inevitably continue to develop and improve itself with the development of science, social progress, and the changes of the times. Therefore, the goal of the digital representation of interior environmental art design is to establish and improve a new design system. The use of digital technology to reflect the state and structure of indoor and outdoor space, the texture of decorative materials, and light and shadow, is shown more realistically in Figure 7.

As grid layouts are used in most grocery and convenience stores, they create a familiar feeling for customers. However, because of this familiarity, it tends to give the customer an on-the-go shopping experience that does not convert traffic into value. Grid layouts are a good option for smaller retailers that have a large inventory, such as toy shops, bookstores, specialty food shops, and home furnishing shops. Support the creation of weak AI intelligence under incomplete information. However, grid floor plans are not optimal for retailers who want to create an upscale branded environment that leads to easy browsing for customers and fails to create a strong impression of the merchandise.

A circular floor plan, sometimes referred to as a runway layout, allows for the best-guided shopping experience. This layout guides the customer through every item on display in the retail shop and is therefore particularly suitable for regional marketing strategies. In a circular plan, the surrounding walls are highly visible and all types of items can be displayed. The loop plan also provides a good basis for a combined layout. With the loop plan, the central part of the retail shop can be set up as a grid or free-flow layout, or even a mixture of the two. The circular floor plan is suitable for most small retail shops, such as clothing and accessories, toys, home furnishings, kitchenware, personal care, and specialty products.

## 5. Conclusion

In today's era of rapid economic and technological development, traditional interior design has completely failed to meet people's current needs, and people need a more comfortable, convenient, intelligent, and accessible lifestyle. With people's high demand for quality of life, artificial intelligence can meet a variety of needs and can be a good solution to the problems of traditional interior design as well as make up for its shortcomings in terms of safety. The cleaning path is made and worked according to the installation route, and the disadvantage is that it cannot be disturbed by the outside world. Artificial intelligence provides favorable conditions for the development of the interior design. Therefore, the application of artificial intelligence in the field of interior design in the big data environment is extremely necessary. In the context of the data era, interior designers can use data thinking to design and use data as a support to guide interior design. Based on behavioral data analysis for interior design, it is important to follow the principle of data shareability as well as to pay attention to user privacy issues in the access to data acquisition. This paper is limited by the technical problems of the equipment, the research is mostly based on theoretical knowledge, and the research is not perfect, so I hope to explore it more deeply in future studies.

## Figures and Tables

**Table 1:** Home matching schemes.

Scenario	TV cabinet	Bed	Bedside table	Dresser	Wardrobe
Scenario 1	TV_1	12	10	dresser_1	12
Scenario 2	TV_2	16	19	17	13
Scenario 3	TV_3	15	15	16	wardrobe_1
Scenario 4	TV_4	bed_149	bedtable_12	10	10
Scenario 5	TV_5	14	13	18	16
Scenario 6	TV_6	11	15	20	20

## Data Availability

The data used to support the findings of this study are available from the author upon request.

## References

[1] Yang L., Yu K., Yang S. X., Chakraborty C., Lu Y., Guo T. (2021). An intelligent trust cloud management method for secure clustering in 5G enabled internet of medical things. *IEEE Transactions on Industrial Informatics*.

[2] Guo Z., Shen Y., Wan S., Shang W., Yu K. (2021). Hybrid intelligence-driven medical image recognition for remote patient diagnosis in internet of medical things. *IEEE Journal of Biomedical and Health Informatics*.

[3] Yu K., Tan L., Mumtaz S. (2021). Securing critical infrastructures: deep-learning-based threat detection in IIoT. *IEEE Communications Magazine*.

[4] Guo Z., Tang C., Niu W. (2017). Fine-grained recommendation mechanism to curb astroturfing in crowdsourcing systems. *IEEE Access*.

[5] Zhang Q., Yu K., Guo Z. (2021). Graph neural networks-driven traffic forecasting for connected internet of vehicles. *IEEE Transactions on Network Science and Engineering*.

[6] Meng D., Xiao Y., Guo Z. (2021). A data-driven intelligent planning model for UAVs routing networks in mobile internet of things. *Computer Communications*.

[7] Ding F., Yu K., Gu Z., Li X., Shi Y. (2022). Perceptual enhancement for autonomous vehicles: restoring visually degraded images for context prediction via adversarial training. *IEEE Transactions on Intelligent Transportation Systems*.

[8] Guo Z., Shen Y., Aloqaily M., Jararweh Y., Yu K. (2021). Probabilistic inference-based modeling for sustainable environmental systems under hybrid cloud infrastructure. *Simulation Modelling Practice and Theory*.

[9] DeLisle J. R., Never B., Grissom T. V. (2020). The big data regime shift in real estate. *Journal of Property Investment & Finance*.

[10] Serrano W. (2018). Digital systems in smart city and infrastructure: digital as a service. *Smart cities*.

[11] Hua H., Wei Z., Qin Y., Wang T., Li L., Cao J (2021). Review of distributed control and optimization in energy internet: from traditional methods to artificial intelligence-based methods. *IET Cyber-Physical Systems: Theory & Applications*.

[12] Rosário A., Moniz L. B., Cruz R. (2021). Data science applied to marketing[J]. *Journal of Information Science and Engineering*.

[13] Hong L., Luo M., Wang R., Lu P., Lu W., Lu L (2018). Big data in health care: applications and challenges. *Data and information management*.

[14] Barbar C., Bass P. D., Barbar R., Bader J., Wondercheck B (2022). Artificial intelligence-driven automation is how we achieve the next level of efficiency in meat processing. *Animal Frontiers*.

[15] van den Homberg M. J. C., Gevaert C. M., Georgiadou Y. (2020). The changing face of accountability in humanitarianism: using artificial intelligence for anticipatory action. *Politics and Governance*.

[16] Zhu Q., Liu Z., Yan J. (2021). Machine learning for metal additive manufacturing: predicting temperature and melt pool fluid dynamics using physics-informed neural networks. *Computational Mechanics*.

[17] Quan S. J., Park J., Economou A., Lee S. (2019). Artificial intelligence-aided design: smart design for sustainable city development. *Environment and Planning B: Urban Analytics and City Science*.

[18] Teles G., Rodrigues J. J. P. C., Rabêlo R. A. L., Kozlov S. A. (2021). Comparative study of support vector machines and random forests machine learning algorithms on credit operation. *Software: Practice and Experience*.

[19] Pesce D., Neirotti P., Paolucci E. (2019). When culture meets digital platforms: value creation and stakeholders’ alignment in big data use. *Current Issues in Tourism*.

[20] Rahmanifard H., Plaksina T. (2019). Application of artificial intelligence techniques in the petroleum industry: a review. *Artificial Intelligence Review*.

[21] Sikora P., Malina L., Kiac M. (2021). Artificial intelligence-based surveillance system for railway crossing traffic. *IEEE Sensors Journal*.

[22] Ahn J. B. (2020). A study on advertising future development roadmap in the fourth industrial revolution era. *International Journal of Internet, Broadcasting and Communication*.

[23] Jablonka K. M., Ongari D., Moosavi S. M., Smit B. (2020). Big-data science in porous materials: materials genomics and machine learning. *Chemical Reviews*.

[24] Zhou H., Xu W., Chen J., Wang W. (2020). Evolutionary V2X technologies toward the internet of vehicles: challenges and opportunities. *Proceedings of the IEEE*.

